# Cerebral Metabolic Rate of Oxygen and Accelerometry‐Based Fatigability in Community‐Dwelling Older Adults

**DOI:** 10.1111/acel.70121

**Published:** 2025-06-13

**Authors:** Emma L. Gay, Caterina Rosano, Paul M. Coen, Nicholaas Bohnen, Theodore Huppert, Yujia (Susanna) Qiao, Nancy W. Glynn

**Affiliations:** ^1^ Department of Epidemiology University of Pittsburgh, School of Public Health Pittsburgh Pennsylvania USA; ^2^ AdventHealth Translational Research Institute Orlando Florida USA; ^3^ Department of Neurology University of Michigan Ann Arbor Michigan USA; ^4^ Department of Electrical and Computer Engineering University of Pittsburgh, Swanson School of Engineering Pittsburgh Pennsylvania USA

**Keywords:** brain energetics, cardiorespiratory fitness, fatigue, physical performance, skeletal muscle energetics

## Abstract

Alterations in energy metabolism may drive fatigue in older age, but prior research primarily focused on skeletal muscle energetics without assessing other systems and utilized self‐reported measures of fatigue. We tested the association between energy metabolism in the brain and an objective measure of fatigability in the Study of Muscle, Mobility and Aging (*N* = 119, age 76.8 ± 4.0 years, 59.7% women). Total brain cerebral metabolic rate of oxygen (CMRO_2_) was measured using arterial spin labeling and T_2_‐relaxation under spin tagging MRI protocols. Accelerometry‐based fatigability status during a fast‐paced 400 m walk was determined using the Pittsburgh Performance Fatigability Index (PPFI, higher = worse). Confounders included skeletal muscle energetics, measured in vivo using spectroscopy and ex vivo using respirometry, cardiorespiratory fitness (VO_2_peak), weight, medication count, and multimorbidity. Multivariable logistic regression models were used to estimate the association (odds ratio (OR)) of CMRO_2_ with PPFI > 0 compared to the referent group PPFI = 0. Models were first adjusted for age and sex and further adjusted for confounders. In this sample, 41.2% had PPFI > 0 (median 3.3% [0.4%–8.0%]). Higher CMRO_2_ was associated with exhibiting performance fatigability (age‐ and sex‐adjusted OR = 1.61, 95% CI: 1.06, 2.45, *p* = 0.03). This association remained significant after adjusting for in vivo skeletal muscle energetics and VO_2_peak, suggesting that performance fatigability is associated with multi‐system impairments in older adults.

## Graphical abstract

We examined the association between whole‐brain energy metabolism and accelerometry‐based performance fatigability in the Study of Muscle, Mobility and Aging. Higher CMRO_2_ was associated with exhibiting performance fatigability (age‐ and sex‐adjusted OR = 1.61, 95% CI: 1.06, 2.45, *p* = 0.03). This association remained significant after adjusting for in vivo skeletal muscle energetics and VO_2_peak, suggesting that performance fatigability is associated with multi‐system impairments in older adults. *Created in BioRender. Gay, E. (2025)* https://BioRender.com/vxe7f7x.

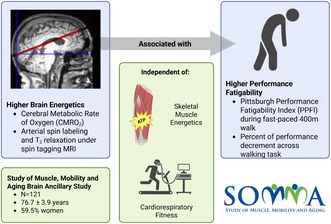

Performance fatigability, the degree one is limited by fatigue during a walking task, is an established measure of impairment in older adults (Enoka et al. 2021; Van Geel et al. 2020) and a subclinical indicator of functional limitations (Schnelle et al. 2012; Hunter 2018; Qiao et al. 2023). Performance fatigability is a subclinical indicator of impending functional decline in older adults (Schrack et al. 2020). The novel Pittsburgh Performance Fatigability Index (PPFI) is a valid, objective, and sensitive measure derived from a walking task designed to mimic real‐world activity (Qiao et al. 2022). We have recently reported that both lower skeletal muscle energetics and cardiorespiratory fitness (CRF, VO_2_peak) were associated with greater accelerometry‐based fatigability in older adults (Qiao et al. 2023, 2024). Although the central nervous system plays an important role in the perception of fatigue (Stults‐Kolehmainen et al. 2020; Marcora 2019; Taylor et al. 2016), studies have primarily assessed neurological patients (Camandola and Mattson 2017; Dalsgaard and Secher 2007; Brooks and Martin 2014; Peralta et al. 2019; Zhang et al. 2018; West et al. 2020) with few reports in older adults without neurological diagnoses (Kato et al. 1999). Studying brain energetics in relation to performance fatigability may help understand the processes underlying this devastating and common phenomenon in older age.

Among the neuroimaging methods to capture brain energetics, T_2_‐relaxation under spin tagging (TRUST) and Arterial Spin Labeling are emerging as non‐invasive approaches to quantify Cerebral Metabolic Rate of Oxygen (CMRO_2_) in a relatively short period of time, with demonstrated validity and reliability, and without contrast agents or radioactive labels (Alsop et al. 2015; Jiang et al. 2021; Xu et al. 2009; Vestergaard et al. 2017). CMRO_2_ reflects the amount of oxygen extracted by the brain parenchyma and increases with age among adults without clinically overt diseases (Xu et al. 2009). Greater CMRO_2_ may indicate greater metabolic costs to maintain homeostasis, perhaps due to reduced cellular efficiency and/or in response to age‐related impairments in other systems (Peng et al. 2014; Lu et al. 2011). A study of patients with multiple sclerosis showed a positive association between CMRO_2_ and perception of fatigue, but there is no evidence in older adults without neurological diseases (West et al. 2020).

We examined the relation between CMRO_2_ and performance fatigability using PPFI (Qiao et al. 2022). We hypothesized that those with higher CMRO_2_ would have greater PPFI. Since muscle energetics and CRF play a critical role in driving fatigability, we assessed to what extent these measures modified the association of CMRO_2_ with fatigability.

We recruited 119 adults (age 76.8 ± 4.0 years, 59.7% women, 90.8% White) from the Study of Muscle, Mobility and Aging with data on CMRO_2_, fatigability, muscle energetics, and CRF (Supplemental Methods; Figure S1). The median PPFI score was 1.3% (range 0%–8.0%) in the full sample, and 41.2% (*n* = 49) had PPFI > 0, a median of 3.3% (range 0.4%–8.0%) (Table 1). Compared to those with PPFI = 0 (*n* = 70), those who exhibited performance fatigability were 2.6 years older and 16.5% more likely to be a woman. Those with PPFI > 0 had 7.9% higher CMRO_2_, 5.9 mL/kg/min lower VO_2_peak, 7.5 kg higher weight, reported taking 1.5 more medications, and were 16.4% more likely to have multimorbidity > 1 compared to those with no fatigability, all *p* < 0.05 (Table 1). Muscle energetics (in vivo and ex vivo), brain atrophy, normalized white matter hyperintensities, the presence of joint pain, and MoCA percentile score > 10% were similar by fatigability status, *p* > 0.05 (Table 1).

**TABLE 1:** Characteristics of participants by performance fatigability status: Study of Muscle, Mobility and Aging (SOMMA)—Brain Ancillary Study.

Variable	No performance fatigability (PPFI = 0) *n* = 70	Exhibiting performance fatigability (PPFI > 0) *n* = 49	*p* *
Age, years	75.7 ± 2.9	78.3 ± 4.7	
Sex, women	37 (52.9)	34 (69.4)	
Cerebral metabolic rate of oxygen (CMRO_2_), μmol/100 g/min	91.1 ± 24.3	99.0 ± 25.2	0.03
ATPmax, mM/s	0.60 ± 0.20	0.53 ± 0.10	0.06
Maximal complex I&II supported OXPHOS, pmol/(s*mg)	71.4 ± 22.7	63.5 ± 16.1	0.1
Maximal electron transport system capacity, pmol/(s*mg)	86.2 ± 25.5	76.8 ± 18.9	0.09
VO_2_peak, mL/kg/min	25.6 ± 5.4	19.7 ± 3.8	< 0.0001
Weight, kg	69.6 ± 12.7	77.1 ± 14.3	< 0.0001
Medication count	4.0 ± 3.3	5.5 ± 3.9	0.02
Multimorbidity > 1	6 (8.6)	12 (25.0)	0.008
Brain atrophy (total gray/intracranial volume)	0.33 ± 0.02	0.33 ± 0.03	0.9
White matter hyperintensities^a^	0.014 ± 0.02	0.018 ± 0.02	0.6
Joint pain (yes/no)	24 (34.3)	15 (30.6)	0.5
MoCA percentile score > 10%	64 (91.4)	45 (91.8)	0.5

*Note:* Values are mean ± standard deviation or *n* (%).

Abbreviations: ATPmax, maximal ATP production; MoCA, Montreal Cognitive Assessment; OXPHOS, oxidative phosphorylation; VO_2_peak, peak oxygen consumption from treadmill cardiopulmonary exercise test.

* Age‐ and sex‐adjusted *p*‐value from ANCOVA (continuous) or logistic regression (categorical).

^a^ Normalized for total white matter.

In age‐ and sex‐adjusted models, higher CMRO_2_ was correlated with a higher PPFI score (*r* = 0.26, *p* = 0.01), lower VO_2_peak (*r* = −0.22, *p* = 0.04), and multimorbidity > 1 (*r* = 0.35, *p* = 0.0008), but not with muscle energetics, weight, or medication counts (Figure S2).

In multivariable models, one SD higher CMRO_2_ (24.9 μmol/100 g/min) was significantly associated with being 61% more likely to exhibit fatigability independent of age and sex (Figure 1). The odds ratio (OR) of CMRO_2_ remained significant after adjusting for ATPmax, VO_2_peak, and weight. Results were similar after adjusting for medication count and multimorbidity, albeit the association was no longer significant, *p* < 0.05. OR for CMRO_2_ only minimally changed after adjusting for either max OXPHOS or max ETS (from 1.61 to 1.52 and 1.50, respectively, Figure 1). Further adjustment for VO_2_peak, weight, medication count, and multimorbidity attenuated the results, *p* > 0.05.

**FIGURE 1:**
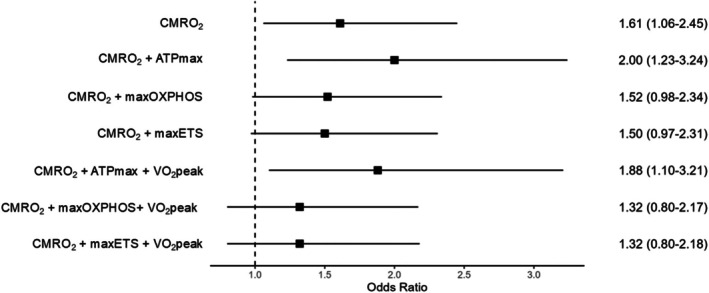
Association between Cerebral Metabolic Rate of Oxygen (CMRO_2_) and Accelerometry‐Based Performance Fatigability. Odds ratios (OR) interpreted as each 1 SD higher CMRO_2_ (24.9 μmol/100 g/min) were associated with higher odds of exhibiting performance fatigability (PPFI > 0) vs. no fatigability (PPFI = 0, reference). All models adjusted for age and sex; models with maxOXPHOS and maxETS additionally adjusted for technician. Abbreviations: ATPmax, maximal ATP production (mM/s); CMRO_2_, cerebral metabolic rate of oxygen (units); maxETS, maximal Complex I and II‐supported ETS (pmol/(s*mg)); maxOXPHOS, maximal Complex I&II supported OXPHOS (pmol/(s*mg)); VO_2_peak, peak oxygen consumption from treadmill cardiopulmonary exercise test.

Our results suggest older adults with higher CMRO_2_ also have higher performance fatigability, independent of age and sex and of varying levels of energetics in other systems. There is an ongoing debate as to whether higher oxygen consumption, irrespective of the tissue or organ being examined, may be compensatory or reflect de‐differentiation. Higher oxygen extraction is associated with higher neural activation and this in turn has been related to less mental fatigue (Darnai et al. 2023). Conversely, higher CMRO_2_ in older age may reflect reduced cellular metabolic efficiency (Peng et al. 2014; Lu et al. 2011), or greater metabolic demands, for example in response to inflammation. A study in patients with multiple sclerosis showed higher CMRO_2_ in relation to fatigue (West et al. 2020); this positive association was interpreted as a response to metabolically active inflammatory processes demanding greater oxygen extraction, and causing worse symptoms and greater fatigue. Inflammation may be central to both higher fatigue and higher CMRO_2_ through the influence of circulating cytokines on the central nervous system (Banks 2005; Yarlagadda et al. 2009) Additionally, neuro‐inflammation is associated with higher energy demands in the brain which could lead to an increase in CMRO_2_ with age (Straub 2017). Thus, as individuals age, neuro‐inflammation may lead to higher CMRO_2_ and symptoms of fatigue/fatigability in older adults.

CMRO_2_ may also increase in response to impairments occurring at the cardiopulmonary level, with lower fitness. In this sample, higher CMRO_2_ was significantly correlated with lower VO_2_peak, and this in turn was associated with higher fatigability. Future studies should assess whether higher CMRO_2_ may be a central response to lower CRF.

Given the role of muscle energetics and CRF on fatigability, we assessed to what extent the relation between CMRO_2_ and PPFI was modified when accounting for these variables. Although the main association remained statistically significant, we also found the influence of muscle energetics varied depending on the metric used. The association between higher CMRO_2_ and fatigability was strengthened (39% higher) when ATPmax was added to the model, whereas it was only minimally attenuated after the addition of ex vivo muscle energetics measures. This may be because ATPmax is an in vivo measure of maximal ATP production during muscle contraction (Coen et al. 2013), whereas respirometry metrics assess mitochondrial function in ideal conditions. Thus, ex vivo measures may not be strong predictors of performance when objective measures of brain energetics, fatigability, and CRF are in the models. Another possibility is due to statistical power.

Participants that exhibited fatigability were 7 kg heavier than those with no fatigability. After adjusting for weight, the relation between CMRO_2_ and fatigability was attenuated. Prior work corroborates our results by revealing an association between higher body mass index and greater perceived physical fatigability in a nationally representative sample of adults aged 60–64 (Cooper et al. 2019). Those exhibiting fatigability took more medications and had more medical conditions than those without fatigability, and this may explain why adjustment for these variables attenuated the association of CRMO_2_ with PPFI.

The SOMMA‐Brain cohort is healthier than the general population, and the majority is White (Rosano et al. 2024) which limits the generalizability of our work. Note that even in our healthier‐than‐average cohort, CMRO_2_ was significantly associated with fatigability after adjusting for in vivo muscle energetics and CRF. Thus, in a less healthy population, the magnitude of the association may be larger than in our findings. Additionally, our analysis examined the relation between CMRO_2_ and performance fatigability in a cognitively normal cohort; thus, this association should be studied in adults with a wider range of cognitive functions given the potential influence on fatigability.

Limitations of our work include our cross‐sectional analysis; thus, we were unable to determine the temporality between CMRO_2_ and fatigability. Future work should evaluate whether changes in CMRO_2_ predict changes in fatigability in older adults, or vice versa. Further, CMRO_2_ was quantified at the whole‐brain level. Thus, metabolic changes in specific regions of the brain may be driving the observed association. While other measures provide regional energy metabolic indices, they present numerous limitations for studies in older adults (Paling et al. 2011). Proton spectroscopy has limited specificity to energy loss, because the metabolites are also linked to parenchymal damage, and perfusion methods require port access which is high risk, especially for older adults. Compared to other methods, our neuroimaging protocol is non‐invasive and requires relatively short scan times (< 15 min). Higher tolerability of the protocol reduces nonparticipation and bias and increases the probability of capturing a more representative sample. Lastly, we cannot exclude the possible measurement errors inherent to TRUST and ASL techniques. For example, we used estimates of blood flow from the Siemens' pulsed‐ASL sequence, optimized for clinical populations, and assumed normal hemoglobin concentrations. These estimates and assumptions may have introduced bias, because older age and sex may influence both transit times and hemoglobin levels. To address this possibility, all our models were adjusted for age and sex.

Our work is strengthened by the use of accelerometry‐based fatigability. PPFI applies weights to emphasize performance decrement at the beginning of the walk and to limit motivation effects at the end of the walk. Further, PPFI considers the entire trajectory of the walk when determining maximum cadence to calculate performance fatigability. This is evidenced by participants reaching their maximal cadence at some point during the walk (Figure S3). Maximal cadence during a typical task performed by older adults may be informative of how performance fatigability may affect their day‐to‐day life. Additional strengths include the measurement of both in vivo and ex vivo measures of muscle energetics and gold‐standard fitness testing.

Emerging studies show brain energy metabolism can respond to behavioral and nutritional interventions in older adults (Zhou et al. 2018; Haeger et al. 2020; Matura et al. 2017; Balestrino and Adriano 2019; Tardy et al. 2020), but their effect on fatigability has not been investigated. Future research should focus on longitudinal associations and the identification of metabolic changes in specific regions of the brain, as it is known that metabolic changes in the prefrontal cortex are associated with fatigue and cognitive function in those with multiple sclerosis (Zuppichini et al. 2023). Additionally, we found that higher perceived physical fatigability was associated with smaller hippocampal, putamen, and thalamus volumes (Wasson et al. 2019). Thus, evaluating regional measures of CMRO_2_ in these locations may identify the metabolic changes behind the observed relation between brain energetics and fatigability in older adults. Our findings indicate brain energetics, skeletal muscle energetics, and cardiorespiratory fitness play a role in performance fatigability in older adults. Collectively, these findings signal that a multi‐symptom approach is needed to better understand the biology of performance fatigability and to develop interventions to reduce fatigability and promote healthy aging.

## Author Contributions

E.L.G., C.R. and N.W.G. had full access to all of the data for the study and took responsibility for the integrity of the data and accuracy of the data analyses. All authors interpreted the data, and critically revised the manuscript for important intellectual content. All authors read and approved the submitted manuscript.

## Conflicts of Interest

The authors declare no conflicts of interest.

## Supporting information


Data S1.

## Data Availability

SOMMA data (May 2024 release) are publicly available by request at https://sommaonline.ucsf.edu/.

## References

[acel70121-bib-0001] Alsop, D. C. , J. A. Detre , X. Golay , et al. 2015. “Recommended Implementation of Arterial Spin‐Labeled Perfusion MRI for Clinical Applications: A Consensus of the ISMRM Perfusion Study Group and the European Consortium for ASL in Dementia.” Magnetic Resonance in Medicine 73: 102–116. 24715426 10.1002/mrm.25197 PMC4190138

[acel70121-bib-0002] Balestrino, M. , and E. Adriano . 2019. “Beyond Sports: Efficacy and Safety of Creatine Supplementation in Pathological or Paraphysiological Conditions of Brain and Muscle.” Medicinal Research Reviews 39: 2427–2459. 31012130 10.1002/med.21590

[acel70121-bib-0003] Banks, W. A. 2005. “Blood‐Brain Barrier Transport of Cytokines: A Mechanism for Neuropathology.” Current Pharmaceutical Design 11: 973–984. 15777248 10.2174/1381612053381684

[acel70121-bib-0004] Brooks, G. A. , and N. A. Martin . 2014. “Cerebral Metabolism Following Traumatic Brain Injury: New Discoveries With Implications for Treatment.” Frontiers in Neuroscience 8: 408. 25709562 10.3389/fnins.2014.00408 PMC4321351

[acel70121-bib-0005] Camandola, S. , and M. P. Mattson . 2017. “Brain Metabolism in Health, Aging, and Neurodegeneration.” EMBO Journal 36: 1474–1492. 28438892 10.15252/embj.201695810 PMC5452017

[acel70121-bib-0006] Coen, P. M. , S. A. Jubrias , G. Distefano , et al. 2013. “Skeletal Muscle Mitochondrial Energetics Are Associated With Maximal Aerobic Capacity and Walking Speed in Older Adults.” Journals of Gerontology. Series A, Biological Sciences and Medical Sciences 68: 447–455. 23051977 10.1093/gerona/gls196 PMC3593613

[acel70121-bib-0007] Cooper, R. , M. Popham , A. J. Santanasto , R. Hardy , N. W. Glynn , and D. Kuh . 2019. “Are BMI and Inflammatory Markers Independently Associated With Physical Fatigability in Old Age?” International Journal of Obesity 43: 832–841. 29795469 10.1038/s41366-018-0087-0 PMC6477893

[acel70121-bib-0008] Dalsgaard, M. K. , and N. H. Secher . 2007. “The Brain at Work: A Cerebral Metabolic Manifestation of Central Fatigue?” Journal of Neuroscience Research 85: 3334–3339. 17394258 10.1002/jnr.21274

[acel70121-bib-0009] Darnai, G. , A. Matuz , H. A. Alhour , et al. 2023. “The Neural Correlates of Mental Fatigue and Reward Processing: A Task‐Based fMRI Study.” NeuroImage 265: 119812. 36526104 10.1016/j.neuroimage.2022.119812

[acel70121-bib-0010] Enoka, R. M. , A. M. Almuklass , M. Alenazy , E. Alvarez , and J. Duchateau . 2021. “Distinguishing Between Fatigue and Fatigability in Multiple Sclerosis.” Neurorehabilitation and Neural Repair 35: 960–973. 34583577 10.1177/15459683211046257

[acel70121-bib-0011] Haeger, A. , A. S. Costa , S. Romanzetti , et al. 2020. “Effect of a Multicomponent Exercise Intervention on Brain Metabolism: A Randomized Controlled Trial on Alzheimer's Pathology (Dementia‐MOVE).” Alzheimer's & Dementia: Translational Research & Clinical Interventions 6: e12032. 32490142 10.1002/trc2.12032 PMC7243943

[acel70121-bib-0012] Hunter, S. K. 2018. “Performance Fatigability: Mechanisms and Task Specificity.” Cold Spring Harbor Perspectives in Medicine 8: a029728. 28507192 10.1101/cshperspect.a029728 PMC6027928

[acel70121-bib-0013] Jiang, D. , S. Deng , C. G. Franklin , et al. 2021. “Validation of T2‐Based Oxygen Extraction Fraction Measurement With 15O Positron Emission Tomography.” Magnetic Resonance in Medicine 85, no. 1: 290–297. 32643207 10.1002/mrm.28410 PMC9973312

[acel70121-bib-0014] Kato, T. , J. Murashita , T. Shioiri , T. Inubushi , and N. Kato . 1999. “Relationship of Energy Metabolism Detected by 31P‐MRS in the Human Brain With Mental Fatigue.” Neuropsychobiology 39: 214–218. 10343187 10.1159/000026587

[acel70121-bib-0015] Lu, H. , F. Xu , K. M. Rodrigue , et al. 2011. “Alterations in Cerebral Metabolic Rate and Blood Supply Across the Adult Lifespan.” Cerebral Cortex 21: 1426–1434. 21051551 10.1093/cercor/bhq224 PMC3097991

[acel70121-bib-0016] Marcora, S. 2019. “Psychobiology of Fatigue During Endurance Exercise.” In Endurance Performance in Sport: Psychological Theory and Interventions, edited by C. Meijen , 15–34. Routledge.

[acel70121-bib-0017] Matura, S. , J. Fleckenstein , R. Deichmann , et al. 2017. “Effects of Aerobic Exercise on Brain Metabolism and Grey Matter Volume in Older Adults: Results of the Randomised Controlled SMART Trial.” Translational Psychiatry 7: e1172. 28934191 10.1038/tp.2017.135 PMC5538117

[acel70121-bib-0018] Paling, D. , X. Golay , C. Wheeler‐Kingshott , R. Kapoor , and D. Miller . 2011. “Energy Failure in Multiple Sclerosis and Its Investigation Using MR Techniques.” Journal of Neurology 258: 2113–2127. 21660561 10.1007/s00415-011-6117-7

[acel70121-bib-0019] Peng, S.‐L. , J. A. Dumas , D. C. Park , et al. 2014. “Age‐Related Increase of Resting Metabolic Rate in the Human Brain.” NeuroImage 98: 176–183. 24814209 10.1016/j.neuroimage.2014.04.078 PMC4099257

[acel70121-bib-0020] Peralta, C. , F. Biafore , T. S. Depetris , and M. Bastianello . 2019. “Recent Advancement and Clinical Implications of 18FDG‐PET in Parkinson's Disease, Atypical Parkinsonisms, and Other Movement Disorders.” Current Neurology and Neuroscience Reports 19: 56. 31256288 10.1007/s11910-019-0966-3

[acel70121-bib-0021] Qiao, Y. S. , J. Harezlak , P. M. Cawthon , et al. 2023. “Validation of the Pittsburgh Performance Fatigability Index in the Study of Muscle, Mobility and Aging (SOMMA).” Journals of Gerontology. Series A, Biological Sciences and Medical Sciences 78, no. 12: 2387–2395. 37566383 10.1093/gerona/glad197 PMC10692427

[acel70121-bib-0022] Qiao, Y. S. , J. Harezlak , K. D. Moored , et al. 2022. “Development of a Novel Accelerometry‐Based Performance Fatigability Measure for Older Adults.” Medicine and Science in Sports and Exercise 54: 1782–1793. 35763596 10.1249/MSS.0000000000002966 PMC9481701

[acel70121-bib-0023] Qiao, Y. S. , A. J. Santanasto , P. M. Coen , et al. 2024. “Associations Between Skeletal Muscle Energetics and Accelerometry‐Based Performance Fatigability: Study of Muscle, Mobility and Aging.” Aging Cell 23: e14015. 37843879 10.1111/acel.14015 PMC11166367

[acel70121-bib-0024] Rosano, C. , L. M. Chahine , E. L. Gay , et al. 2024. “Higher Striatal Dopamine Is Related With Lower Physical Performance Fatigability in Community‐Dwelling Older Adults.” Journals of Gerontology. Series A, Biological Sciences and Medical Sciences 79: glae209. 39208421 10.1093/gerona/glae209 PMC11447735

[acel70121-bib-0025] Schnelle, J. F. , M. S. Buchowski , T. A. Ikizler , D. W. Durkin , L. Beuscher , and S. F. Simmons . 2012. “Evaluation of Two Fatigability Severity Measures in Elderly Adults.” Journal of the American Geriatrics Society 60: 1527–1533. 22860899 10.1111/j.1532-5415.2012.04062.x PMC3419324

[acel70121-bib-0026] Schrack, J. A. , E. M. Simonsick , and N. W. Glynn . 2020. “Fatigability: A Prognostic Indicator of Phenotypic Aging.” Journals of Gerontology. Series A, Biological Sciences and Medical Sciences 75: e63–e66. 32936913 10.1093/gerona/glaa185 PMC7749190

[acel70121-bib-0027] Straub, R. H. 2017. “The Brain and Immune System Prompt Energy Shortage in Chronic Inflammation and Ageing.” Nature Reviews Rheumatology 13: 743–751. 29021568 10.1038/nrrheum.2017.172

[acel70121-bib-0028] Stults‐Kolehmainen, M. A. , M. Blacutt , J. B. Bartholomew , et al. 2020. “Motivation States for Physical Activity and Sedentary Behavior: Desire, Urge, Wanting, and Craving.” Frontiers in Psychology 11: 568390. 33240154 10.3389/fpsyg.2020.568390 PMC7677192

[acel70121-bib-0029] Tardy, A.‐L. , E. Pouteau , D. Marquez , C. Yilmaz , and A. Scholey . 2020. “Vitamins and Minerals for Energy, Fatigue and Cognition: A Narrative Review of the Biochemical and Clinical Evidence.” Nutrients 12: 228. 31963141 10.3390/nu12010228 PMC7019700

[acel70121-bib-0030] Taylor, J. L. , M. Amann , J. Duchateau , R. Meeusen , and C. L. Rice . 2016. “Neural Contributions to Muscle Fatigue: From the Brain to the Muscle and Back Again.” Medicine and Science in Sports and Exercise 48: 2294–2306. 27003703 10.1249/MSS.0000000000000923 PMC5033663

[acel70121-bib-0031] Van Geel, F. , L. Moumdjian , I. Lamers , H. Bielen , and P. Feys . 2020. “Measuring Walking‐Related Performance Fatigability in Clinical Practice: A Systematic Review.” European Journal of Physical and Rehabilitation Medicine 56: 88–103. 31742368 10.23736/S1973-9087.19.05878-7

[acel70121-bib-0032] Vestergaard, M. B. , U. Lindberg , N. J. Aachmann‐Andersen , et al. 2017. “Comparison of Global Cerebral Blood Flow Measured by Phase‐Contrast Mapping MRI With 15 O‐H_2_O Positron Emission Tomography.” Journal of Magnetic Resonance Imaging 45: 692–699. 27619317 10.1002/jmri.25442 PMC5324556

[acel70121-bib-0033] Wasson, E. , A. L. Rosso , A. J. Santanasto , et al. 2019. “Neural Correlates of Perceived Physical and Mental Fatigability in Older Adults: A Pilot Study.” Experimental Gerontology 115: 139–147. 30528639 10.1016/j.exger.2018.12.003 PMC6331252

[acel70121-bib-0034] West, K. , D. Sivakolundu , G. Maruthy , et al. 2020. “Baseline Cerebral Metabolism Predicts Fatigue and Cognition in Multiple Sclerosis Patients.” NeuroImage: Clinical 27: 102281. 32544855 10.1016/j.nicl.2020.102281 PMC7298673

[acel70121-bib-0035] Xu, F. , Y. Ge , and H. Lu . 2009. “Noninvasive Quantification of Whole‐Brain Cerebral Metabolic Rate of Oxygen (CMRO2) by MRI.” Magnetic Resonance in Medicine 62: 141–148. 19353674 10.1002/mrm.21994 PMC2726987

[acel70121-bib-0036] Yarlagadda, A. , E. Alfson , and A. H. Clayton . 2009. “The Blood Brain Barrier and the Role of Cytokines in Neuropsychiatry.” Psychiatry 6: 18–22. PMC2801483 20049146

[acel70121-bib-0037] Zhang, L. , T. Li , Y. Yuan , et al. 2018. “Brain Metabolic Correlates of Fatigue in Parkinson's Disease: A PET Study.” International Journal of Neuroscience 128: 330–336. 28918694 10.1080/00207454.2017.1381093

[acel70121-bib-0038] Zhou, M. , H. Liao , L. P. Sreepada , J. R. Ladner , J. A. Balschi , and A. P. Lin . 2018. “Tai Chi Improves Brain Metabolism and Muscle Energetics in Older Adults.” Journal of Neuroimaging 28: 359–364. 29667260 10.1111/jon.12515 PMC6055800

[acel70121-bib-0039] Zuppichini, M. D. , D. K. Sivakolundu , K. L. West , D. T. Okuda , and B. Rypma . 2023. “Investigating the Link Between Regional Oxygen Metabolism and Cognitive Speed in Multiple Sclerosis: Implications for Fatigue.” Multiple Sclerosis and Related Disorders 80: 105074. 37866021 10.1016/j.msard.2023.105074

