# Supplementary material for: Cerebral Metabolic Rate of Oxygen and Accelerometry‐Based Fatigability in Community‐Dwelling Older Adults
Source: Aging Cell. 2025 Jun 13;24(8):e70121. doi: 10.1111/acel.70121 (PMC12341810; doi:10.1111/acel.70121)
Supplement: Supplementary file 1 — Data S1. [file ACEL-24-e70121-s001.pdf]

SUPPORTING INFORMATION FOR:

Cerebral Metabolic Rate of Oxygen and Accelerometry-Based Performance Fatigability in Community-Dwelling Older Adults

Emma L. Gay, MPH*[1], Caterina Rosano, MD, MPH*[1], Paul M. Coen, PhD[2], Nicholaas Bohnen, MD, PhD[3], Theodore Huppert, PhD[4], Yujia (Susanna) Qiao, PhD[1], and Nancy W. Glynn, PhD[1]

*Contributed equally

[1]University of Pittsburgh, School of Public Health, Department of Epidemiology, Pittsburgh, PA

[2]AdventHealth, Translational Research Institute, Orlando, FL

[3]University of Michigan, Department of Neurology, Ann Arbor, MI

[4]University of Pittsburgh, Swanson School of Engineering, Department of Electrical and Computer Engineering, Pittsburgh, PA

Corresponding Author:

Caterina Rosano, MD, MPH
University of Pittsburgh, School of Public Health, Department of Epidemiology
130 DeSoto Street, 5139 Public Health
Pittsburgh, PA 15261
rosanoc@edc.pitt.edu

Supplemental Methods

Study Sample

Older adults age ≥70 years enrolled in the Study of Muscle, Mobility and Aging (SOMMA, http://sommaonline.ucsf.edu) from the Pittsburgh clinical site (N=439) were recruited for the SOMMA-Brain Ancillary study. Exclusion criteria for the parent study included: inability to walk one-quarter of a mile or climb a flight of stairs; body mass index (BMI) ≥ 40 kg/m^2; active malignancy or dementia; or medical contraindication to biopsy or magnetic resonance imaging (MRI). Additionally, participants had to be able to complete a usual-paced 400m walk (Cummings et al. 2023). To be eligible for the SOMMA-Brain Ancillary study the skeletal muscle biopsy had to have occurred within the past 12 months (n=285), and without diagnosed neurologic disorder. A total of 150 individuals agreed to participate in the ancillary study and completed the neuroimaging protocol (Supplemental Figure 1) (Rosano et al. 2024). Of those, 26 did not complete the walking test needed to derive PPFI (19 were not tested due to scheduling issues, 2 could not be reached, 2 unable to finish walk, 1 technical error, 1 was ineligible, and 1 refused). Of the 124, 5 participants did not have useable accelerometry data for deriving the performance fatigability outcome. Thus, the final analytic sample was n=119 (Figure 1). The average time between muscle biopsy and neuroimaging was 9.4 months. The WIRB-Copernicus Group Institutional Review Board (IRB# 20180764) and the University of Pittsburgh Human Research Protection Office (PittPRO# 20110230) approved the study and all participants gave informed written consent.

Performance Fatigability

Participants wore an ActiGraph GT9X accelerometer (ActiGraph LLC, Pensacola, FL) on both ankles during the fast-paced 400m walk. Triaxial raw accelerometer data were collected at a

sampling frequency of 100Hz. During the fast-paced 400m walk, participants were instructed to walk as quickly as possible, without running, at a pace they could maintain for 10 laps on a 20m course (Simonsick et al. 2006).

Raw accelerometer data from the non-dominant ankle were processed in R to calculate the Pittsburgh Performance Fatigability Index (PPFI) (Qiao et al. 2022), a ratio comparing the area under the individual's observed cadence-versus-time trajectory during the walk to a hypothetical area that would be observed if the individual's maximum cadence were maintained throughout the walk. Individual-level smoothed cadence trajectories were fit using penalized regression splines. Specific details about the derivation of PPFI have been published (Qiao et al. 2022). Higher PPFI score (range 0-100%) indicates greater performance fatigability (Qiao et al. 2022). Participants who completed the fast-paced 400m walking within 5 minutes exhibited no performance fatigability during the walking task and thus, were classified as PPFI=0 (Qiao et al. 2022). PPFI was initially validated at the non-dominant wrist, but the non-dominant ankle was an appropriate substitution as identification of physical activity is 95% accurate for ankle worn devices (Mannini et al. 2013). Additionally, stride segmentation (i.e., cadence), which is an essential input to derive PPFI, is highly accurate for ankle worn accelerometry when using the ADEPT R package with estimated deviations for stride of 1.24% at the left ankle and 1.30% at the right ankle (Karas et al. 2021).

Cortical volume and white matter hyperintensity quantitation.

MRI volumes were distortion-corrected, registered, and segmented as described in (Glasser et al. 2013) using a combination of FSL and FreeSurfer analysis programs. The T1-weighted (MPRAGE) was collected at a 0.8mm isotropic resolution (320x320x192) (TR=2400ms, TE=2.24ms, TI=1000ms, FA=8º). Total and subcortical gray matter volumes were

obtained via FreeSurfer (Dale et al. 1999). Regions were labeled with reference to the Desikan atlas (Desikan et al. 2006). Gray matter atrophy was calculated as total gray matter volume/intracranial volume and subcortical gray matter atrophy as subcortical gray matter volume/intracranial volume. White matter hyperintensity (WMH) on MRI, a measure of cSVD (cerebral small vessel disease), was quantitated via automated segmentation methods as previously described (Schmidt & Wink 2017) Using a turbo-spin echo T2w-FLAIR MRI volume. The FLAIR volume was collected at 0.5 x 0.5 x 3.0mm (512 x 512 x 48) (TR=9160ms, TE=90ms, TI=2520ms, FA = 150°).

Brain MRI - Cerebral Metabolic Rate of Oxygen ($CMRO_2$)

$CMRO_2$, the amount of oxygen consumed per unit mass and per unit time, depends on cerebral blood flow (CBF) and oxygen extraction fraction (OEF)(Xu et al. 2009). The Fick principle of arteriovenous oxygen difference provides an absolute measure of $CMRO_2$ in the whole brain (Kety & Schmidt 1945; Lee et al. 2013). OEF, reflecting the proportion of O_2 extracted from the blood, was estimated non-invasively via T_2-relaxation under spin tagging MRI in the sagittal sinus and arterial oxygen saturation via pulse oximetry (Jiang et al. 2021). CBF, reflecting the supply of O_2 to the brain, was assessed using arterial spin labeling perfusion MRI (Siemens Biograph mMR PET/MR)(Alsop et al. 2015).

Cerebral blood flow was obtained from the Siemens' pulsed arterial spin labeling (PASL, QQUIPSS-II) clinical sequence. The scan resolution was 4x4x8mm (64x64x9) with a 10mm axial slice gap (TR=2500ms, TE=11ms, FA=90°, TI = 700ms/1800ms). The Siemens derived perfusion map computed over the average of the 91 TRs (227.7s) scan was used for analysis. The M0 EPI volume was used to linear register to the same session anatomical T1w volume. Global

ASL perfusion was computed as the average over the cortical regions labeled from the Desikan atlas using FreeSurfer as described in the previous section.

Oxygen extraction fraction was obtained from the MRI TRUST methods (Lu et al. 2012). First, a three-dimensional phase contrast angiography structural scan was collected with a velocity encode factor (VENC) of 30cm/s to identify the dural sinus blood vessels. The single-slice TRUST image was positioned according to the location of the confluence of sinuses visually identified in the angiography image at the time of MRI acquisition. The TRUST slice has a resolution of 3.43 x 3.43 x 5.0mm (64x64x1) (TR=3000ms, TI=1020ms; FA=90°) with 24 echoes 18ms to 84ms with a 3ms step. The angiography image was then used to verify the selection of the confluence of sinuses as the region-of-interest in TRUST analysis. The T_2 relaxation from the multi-echo TRUST data was used to estimate oxygen extraction fractions using equations 1-4 from (Lu et al. 2012) given by:

$$\frac{1}{T_2} = A + B \cdot (1 - Y) + C \cdot (1 - Y)^2$$

$$A = a_0 + a_1 \cdot Hct + a_2 \cdot Hct^2$$

$$B = b_1 \cdot Hct + b_2 \cdot Hct^2$$

$$C = c_1 \cdot Hct \cdot (1 - Hct)$$

Where the coefficients used were based on calibration studies at the 3T field strength ($\tau_{CPMG} = 10ms$) used in this study (a_0 = -13.5 s^{-1}, a_1 = 80.2 s^{-1}, a_2 = -75.9 s^{-1}, b_1 = -0.5 s^{-1}, b_2 = 3.4 s^{-1} , c_1 = 247.4 s^{-1}. Data with a R^2 (goodness-of-fit) <0.97 were removed. The global estimate of $CMRO_2$ was then computed from the product of global blood flow and OEF (1-Y) using the equation:

$$CMRO2 = (1-Y) * CBF * Hb / 64.5kg/mole * 4\ O_2/Hb$$

Where Hb is the concentration of hemoglobin in blood, 64.5 kg/mole is the molecular weight of the hemoglobin complex, and the hemoglobin complex carries four oxygen molecules. Since hemoglobin concentration was not measured in this study, and participants did not have clinical signs of anemia, a value of 14 gm/dL for hemoglobin was used, covarying for age and sex in all models.

Skeletal Muscle Energetics

Maximal ATP Production**:** Based on Qiao et al. finding that lower maximal ATP production (ATPmax) was associated with higher PPFI during a usual-paced 400m walk in the SOMMA parent study (Qiao et al. 2024), we examined this measure as a potential covariate in our models. ATPmax was quantified using ^{31}P magnetic resonance spectroscopy to measure the rate of phosphocreatine (PCr) regeneration following a short bout of exercise. A 3 Tesla MRI scanner (Siemens Medical System – Prisma) using a 12” dual-tuned, surface radiofrequency coil (PulseTeq, Limited) placed over the right distal vastus lateralis was used to collect ^{31}P spectra. Participants performed two bouts of isometric knee extension against the resistance of an ankle strap as previously described (Cummings et al. 2023). PCr recovery rate after exercise was fit and the time-constant of the mono-exponential fit (tau) was used to calculate ATPmax (Blei et al. 1993; Jubrias et al. 2003; Amara et al. 2008). In SOMMA, the mean coefficient of variation for duplicate measures of ATPmax was 9.9% across clinic sites (Mau et al. 2023).

Skeletal Muscle Respiration**:** Our previous work in SOMMA also revealed that lower maximal complex I & II supported oxidative phosphorylation (max OXPHOS) and maximal electron transport system (max ETS) were associated with higher PPFI during a usual-paced 400m walk (Qiao et al. 2024), thus we also evaluated these two measures of skeletal muscle respiration as potential covariates. A skeletal muscle biopsy was taken from the medial vastus

lateralis after a 12-hour fast and limited exercise for 48 hours prior to the procedure (Zamora et al. 2024). Approximately 20mg of the specimen was placed in a biopsy preserving solution for high-resolution respirometry (Zamora et al. 2024). Approximately 2-3 mg of myofiber bundles were then weighed and placed into Oxygraph-2K respirometer chambers (O2K, Oroboros Instruments, Austria). Assays were run in duplicate at 37°C within a specific range of O_2 concentrations (400-200 μm). Steady-state oxygen flux was normalized to the fiber bundle wet weight using Datlab 7.4 software (Coen et al. 2013; Mau et al. 2023). Technician was controlled for in analysis.

Cardiorespiratory Fitness

Cardiorespiratory fitness was measured by cardiopulmonary exercise testing (CPET) using a modified symptom-limited Balke treadmill protocol where speed and grade increased incrementally (Wolf et al. 2024). After a 5-minute preferred walking speed treadmill task, testing for VO_2peak began with incremental rate (0.5 mph) and/or slope (2.5%) increases in 2 minute stages until respiratory exchange ratio was $\geq$ 1.05 and Borg Rating of Perceived Exertion was $\geq$17. Absolute VO_2peak was determined in the BREEZESUITE software as the highest 30-second average of VO_2 (mL/min) achieved (Wolf et al. 2024). Both absolute and weight adjusted (mL/kg/min) VO_2peak were used in analyses as appropriate.

Covariates

Age in years, brain atrophy, white matter hyperintensities and joint pain in the last month were measured during the baseline MRI visit of SOMMA-Brain as previously described (Rosano et al. 2024). Measures collected during the SOMMA baseline visit of the parent study included sex, race, and weight measured using a balance beam or digital scale, without shoes and with light clothing. Participants reported the prescription medications they had taken in the past 30

days, a count of medications was used in this analysis. Self-reported history of physician diagnosed chronic health conditions and depressive symptoms were combined to create the SOMMA multimorbidity index, which was dichotomized to 0-1 and >1 for this analysis. The Montreal Cognitive Assessment was performed during SOMMA baseline day 1. Scores were reported as a percentile adjusted for age, sex, race, and education (0-100)(Sachs et al. 2022)

Statistical Analysis

Characteristics of the participants by performance fatigability status (PPFI=0 vs PPFI>0) were compared using ANCOVA (continuous) or logistic regression (categorical); reported p-values reflect age and sex adjustment. We examined associations between $CMRO_2$, PPFI (continuous), and variables of interest that were significantly different by performance fatigability status using partial Pearson and Spearman (PPFI) correlations adjusted for age and sex. Scatterplots were examined for non-linear associations between $CMRO_2$ and PPFI as well as $CMRO_2$ and skeletal muscle energetics and VO_2peak. Logistic regression was used to examine the association between $CMRO_2$ and performance fatigability status adjusted for age, sex, plus skeletal muscle energetics (and technician for ex vivo measures) in separate models. Next, we adjusted for cardiorespiratory fitness, weight; and last, for any other variables that were significantly different by performance fatigability status. The units of the explanatory variable, $CMRO_2$, were scaled to one standard deviation (SD) for interpretation, Additionally, ATPmax, max OXPHOS, max ETS and VO2peak were entered into models as standardized variables. Analyses were conducted in SAS version 9.4 using the May 2024 SOMMA data release.

References

Alsop DC, Detre JA, Golay X, Günther M, Hendrikse J, Hernandez-Garcia L, Lu H, MacIntosh BJ, Parkes LM, Smits M, van Osch MJP, Wang DJJ, Wong EC & Zaharchuk G (2015) Recommended implementation of arterial spin-labeled perfusion MRI for clinical applications: A consensus of the ISMRM perfusion study group and the European consortium for ASL in dementia. *Magn. Reson. Med.* 73, 102–116.

Amara CE, Marcinek DJ, Shankland EG, Schenkman KA, Arakaki LSL & Conley KE (2008) Mitochondrial function in vivo: spectroscopy provides window on cellular energetics. *Methods* 46, 312–318.

Blei ML, Conley KE & Kushmerick MJ (1993) Separate measures of ATP utilization and recovery in human skeletal muscle. *J Physiol (Lond)* 465, 203–222.

Coen PM, Jubrias SA, Distefano G, Amati F, Mackey DC, Glynn NW, Manini TM, Wohlgemuth SE, Leeuwenburgh C, Cummings SR, Newman AB, Ferrucci L, Toledo FGS, Shankland E, Conley KE & Goodpaster BH (2013) Skeletal muscle mitochondrial energetics are associated with maximal aerobic capacity and walking speed in older adults. *J. Gerontol. A Biol. Sci. Med. Sci.* 68, 447–455.

Cummings SR, Newman AB, Coen PM, Hepple RT, Collins R, Kennedy Ms K, Danielson M, Peters K, Blackwell T, Johnson E, Mau T, Shankland EG, Lui L-Y, Patel S, Young D, Glynn NW, Strotmeyer ES, Esser KA, Marcinek DJ, Goodpaster BH, Kritchevsky S & Cawthon PM (2023) The Study of Muscle, Mobility and Aging (SOMMA): A Unique Cohort Study About the Cellular Biology of Aging and Age-related Loss of Mobility. *J. Gerontol. A Biol. Sci. Med. Sci.* 78, 2083–2093.

Dale AM, Fischl B & Sereno MI (1999) Cortical surface-based analysis. I. Segmentation and surface reconstruction. *Neuroimage* 9, 179–194.

Desikan RS, Ségonne F, Fischl B, Quinn BT, Dickerson BC, Blacker D, Buckner RL, Dale AM, Maguire RP, Hyman BT, Albert MS & Killiany RJ (2006) An automated labeling system for subdividing the human cerebral cortex on MRI scans into gyral based regions of interest. *Neuroimage* 31, 968–980.

Glasser MF, Sotiropoulos SN, Wilson JA, Coalson TS, Fischl B, Andersson JL, Xu J, Jbabdi S, Webster M, Polimeni JR, Van Essen DC, Jenkinson M & WU-Minn HCP Consortium (2013) The minimal preprocessing pipelines for the Human Connectome Project. *Neuroimage* 80, 105–124.

Jiang D, Deng S, Franklin CG, O'Boyle M, Zhang W, Heyl BL, Pan L, Jerabek PA, Fox PT & Lu H (2021) Validation of T2 -based oxygen extraction fraction measurement with 15 O positron emission tomography. *Magn. Reson. Med.* 85, 290–297.

Jubrias SA, Crowther GJ, Shankland EG, Gronka RK & Conley KE (2003) Acidosis inhibits oxidative phosphorylation in contracting human skeletal muscle in vivo. *J Physiol (Lond)* 553, 589–599.

Karas M, Stra Czkiewicz M, Fadel W, Harezlak J, Crainiceanu CM & Urbanek JK (2021) Adaptive empirical pattern transformation (ADEPT) with application to walking stride segmentation. *Biostatistics* 22, 331–347.

Kety SS & Schmidt CF (1945) The determination of cerebral blood flow in man by the use of nitrous oxide in low concentrations. *American Journal of Physiology-Legacy Content* 143, 53–66.

Lee JJ, Powers WJ, Faulkner CB, Boyle PJ & Derdeyn CP (2013) The Kety-Schmidt technique for quantitative perfusion and oxygen metabolism measurements in the MR imaging environment. *AJNR Am J Neuroradiol* 34, E100-2.

Lu H, Xu F, Grgac K, Liu P, Qin Q & van Zijl P (2012) Calibration and validation of TRUST MRI for the estimation of cerebral blood oxygenation. *Magn. Reson. Med.* 67, 42–49.

Mannini A, Intille SS, Rosenberger M, Sabatini AM & Haskell W (2013) Activity recognition using a single accelerometer placed at the wrist or ankle. *Med. Sci. Sports Exerc.* 45, 2193–2203.

Mau T, Lui L-Y, Distefano G, Kramer PA, Ramos SV, Toledo FGS, Santanasto AJ, Shankland EG, Marcinek DJ, Jurczak MJ, Sipula I, Bello FM, Duchowny KA, Molina AJA, Sparks LM, Goodpaster BH, Hepple RT, Kritchevsky SB, Newman AB, Cawthon PM, Cummings SR & Coen PM (2023) Mitochondrial energetics in skeletal muscle are associated with leg power and cardiorespiratory fitness in the study of muscle, mobility and aging. *J. Gerontol. A Biol. Sci. Med. Sci.* 78, 1367–1375.

Qiao YS, Harezlak J, Moored KD, Urbanek JK, Boudreau RM, Toto PE, Hawkins M, Santanasto AJ, Schrack JA, Simonsick EM & Glynn NW (2022) Development of a Novel Accelerometry-Based Performance Fatigability Measure for Older Adults. *Med. Sci. Sports Exerc.* 54, 1782–1793.

Qiao YS, Santanasto AJ, Coen PM, Cawthon PM, Cummings SR, Forman DE, Goodpaster BH, Harezlak J, Hawkins M, Kritchevsky SB, Nicklas BJ, Toledo FGS, Toto PE, Newman AB & Glynn NW (2024) Associations between skeletal muscle energetics and accelerometry-based performance fatigability: Study of Muscle, Mobility and Aging. *Aging Cell* 23, e14015.

Rosano C, Chahine LM, Gay EL, Coen PM, Bohnen NI, Studenski SA, LoPresti B, Rosso AL, Huppert T, Newman AB, Royse SK, Kritchevsky SB & Glynn NW (2024) Higher Striatal Dopamine is Related With Lower Physical Performance Fatigability in Community-Dwelling Older Adults. *J. Gerontol. A Biol. Sci. Med. Sci.* 79.

Sachs BC, Chelune GJ, Rapp SR, Couto AM, Willard JJ, Williamson JD, Sink KM, Coker LH, Gaussoin SA, Gure TR, Lerner AJ, Nichols LO, Still CH, Wadley VG & Pajewski NM (2022) Robust demographically-adjusted normative data for the Montreal Cognitive Assessment (MoCA): Results from the systolic blood pressure intervention trial. *Clin. Neuropsychol.* 36, 2237–2259.

Schmidt P & Wink L (2017) LST: A lesion segmentation tool for SPM. *Manual/Documentation for version 3.0.0 October 2019*.

Simonsick EM, Fan E & Fleg JL (2006) Estimating cardiorespiratory fitness in well-functioning older adults: treadmill validation of the long distance corridor walk. *J. Am. Geriatr. Soc.* 54, 127–132.

Wolf C, Blackwell TL, Johnson E, Glynn NW, Nicklas B, Kritchevsky SB, Carnero EA, Cawthon PM, Cummings SR, Toledo FGS, Newman AB, Forman DE & Goodpaster BH (2024) Cardiopulmonary exercise testing in a prospective multicenter cohort of older adults. *Med. Sci. Sports Exerc.*

Xu F, Ge Y & Lu H (2009) Noninvasive quantification of whole-brain cerebral metabolic rate of oxygen (CMRO2) by MRI. *Magn. Reson. Med.* 62, 141–148.

Zamora Z, Lui L-Y, Sparks LM, Justice J, Lyles M, Gentle L, Gregory H, Yeo RX, Kershaw EE, Stefanovic-Racic M, Newman AB, Kritchevsky S & Toledo FGS (2024) Percutaneous biopsies of skeletal muscle and adipose tissue in individuals older than 70: methods and outcomes in the Study of Muscle, Mobility and Aging (SOMMA). *Geroscience* 46, 3419–3428.

Supplemental Figure 1: Flowchart for Inclusion and Exclusion of Participants from the Study of Muscle, Mobility and Aging (SOMMA) – Brain Ancillary Study in this Analysis

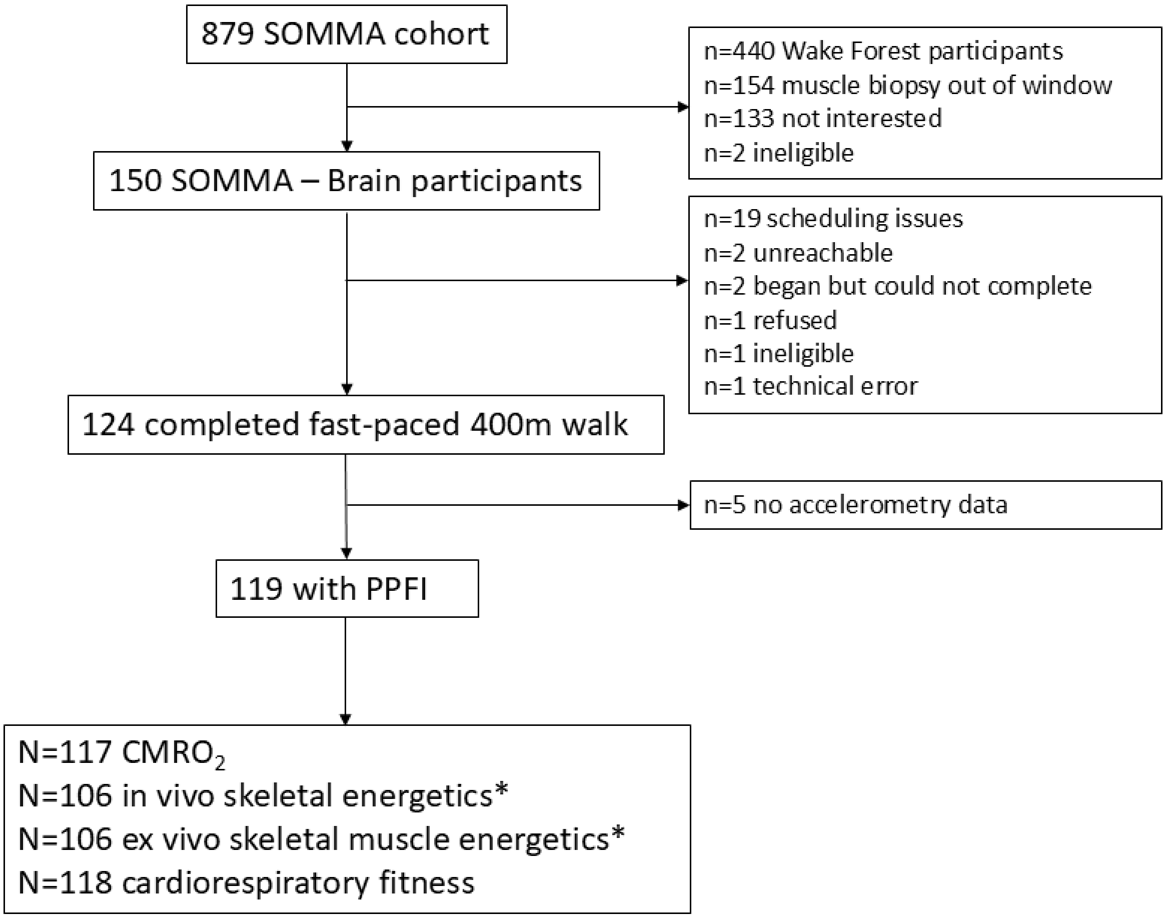

*different composition of participants

Supplemental Figure 2: Partial Pearson and Spearman Correlations for the Pittsburgh Fatigability Index (PPFI) and Main Contributors of PPFI

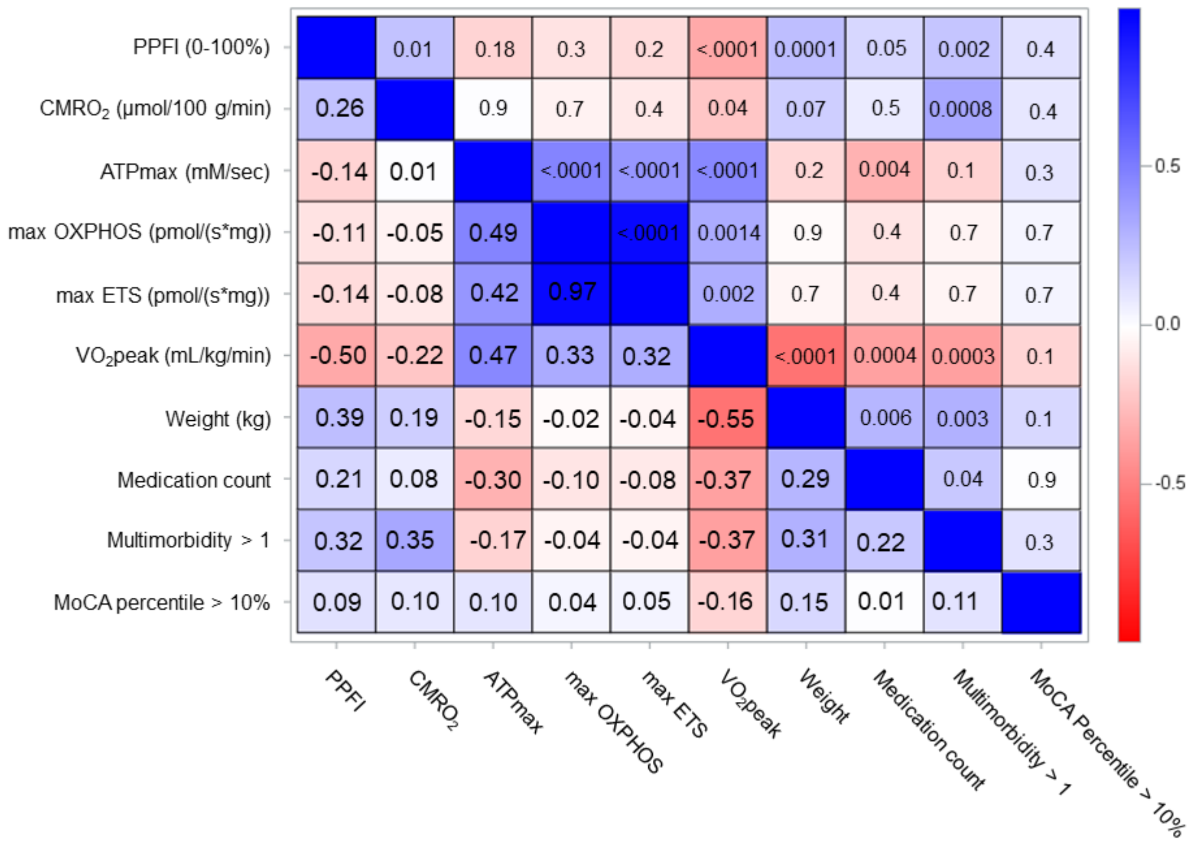

Partial correlations adjusted for age and sex. Pearson: $CMRO_2$, ATPmax, max OXPHOS, max ETS, VO_2peak, weight, medication count, multimorbidity>1, MoCA percentile score>10%; Spearman: PPFI

Bottom left: correlation coefficients (r)
Top right: p-value

Abbreviations: PPFI – Pittsburgh Performance Fatigability Index; $CMRO_2$ – Cerebral Metabolic Rate of Oxygen; OXPHOS – oxidative phosphorylation; ETS – electron transport system; MoCA – Montreal Cognitive Assessment

Supplemental Figure 3: Individual Cadence-vs-Time Trajectories from the Fast-Paced 400m Walk

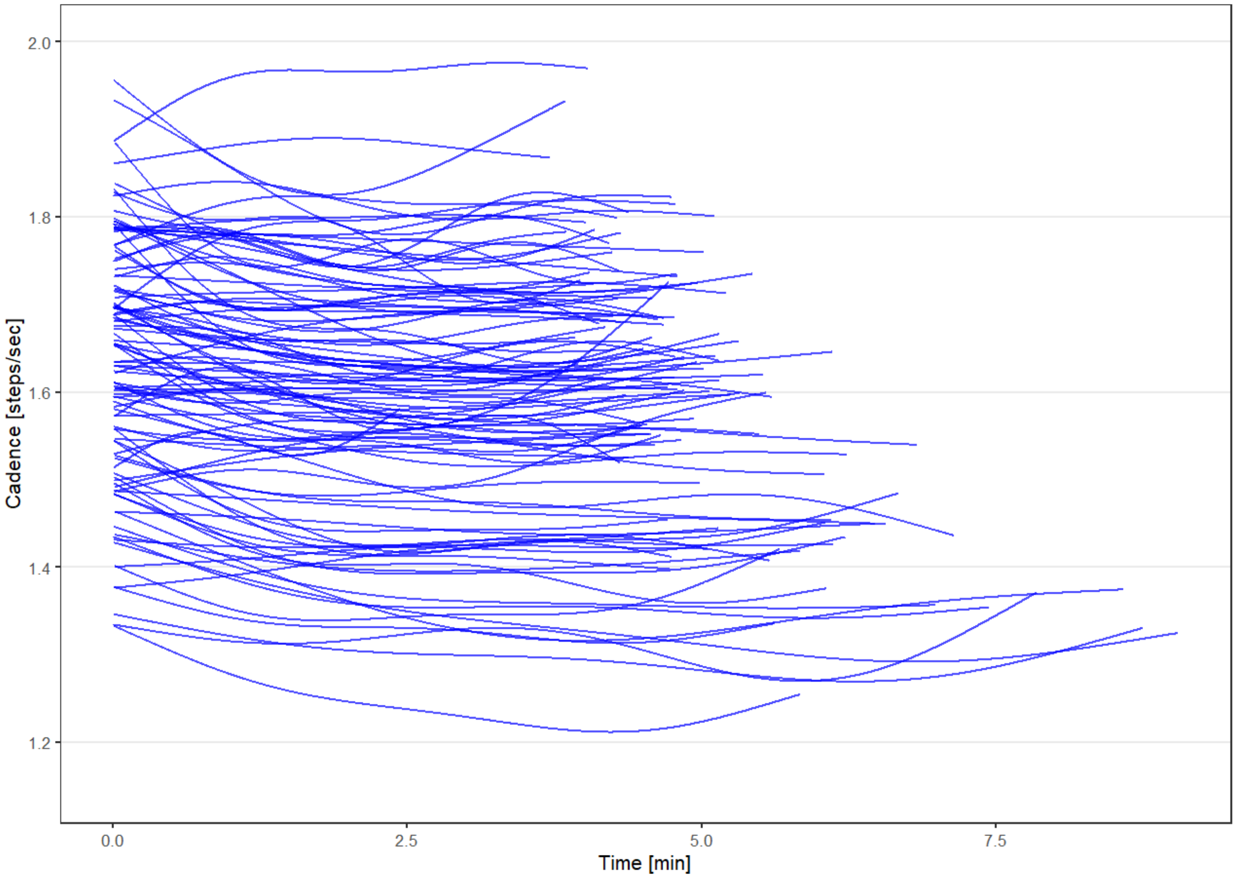

Participants generally followed one of the following pacing strategies during the fast-400m walk: (1) slowing down consistently across the walk, (2) speeding up consistently across the walk, and (3) slowing down at the beginning and speeding up at the end.
